# Sex differences in postsurgical skeletal muscle depletion after donation of living-donor liver transplantation, although minimal, should not be ignored

**DOI:** 10.1186/s12893-020-00781-0

**Published:** 2020-06-03

**Authors:** Rihito Nagata, Nobuhisa Akamatsu, Akiko Nakazawa, Junichi Kaneko, Takeaki Ishizawa, Junichi Arita, Kiyoshi Hasegawa

**Affiliations:** Artificial Organ and Transplantation Division, Department of Surgery, Graduate School of Medicine, The University of Tokyo, 7-3-1, Hongo, Bunkyo-ku, Tokyo, 113-8655 Japan

**Keywords:** Living-donor liver transplantation, Psoas major muscle, Skeletal muscle depletion, Sex difference

## Abstract

**Background:**

Donor safety is the top priority in living-donor liver transplantation. Splenic hypertrophy and platelet count decrease after donor surgery are reported to correlate with the extent of hepatectomy, but other aftereffects of donor surgeries are unclear. In this study, we evaluated the surgical effects of donor hepatectomy on skeletal muscle depletion and their potential sex differences.

**Methods:**

Among a total of 450 consecutive donor hepatectomies performed from April 2001 through March 2017, 277 donors who completed both preoperative and postoperative (60–119 days postsurgery) evaluation by computed tomography were the subjects of this study. Donors aged 45 years or older were considered elderly donors. Postoperative skeletal muscle depletion was assessed on the basis of the cross-sectional area of the psoas major muscle. Postoperative changes in the spleen volume and platelet count ratios were also analysed to evaluate the effects of major hepatectomy.

**Results:**

The decrease in the postoperative skeletal muscle mass in the overall donor population was slight (99.4 ± 6.3%). Of the 277 donors, 59 (21.3%) exhibited skeletal muscle depletion (i.e., < 95% of the preoperative value). Multivariate analysis revealed that elderly donor (OR:2.30, 95% C.I.: 1.27–4.24) and female donor (OR: 1.94, 95% C.I. 1.04–3.59) were independent risk factors for postoperative skeletal muscle depletion. Stratification of the subjects into four groups by age and sex revealed that the elderly female donor group had significantly less skeletal muscle mass postoperatively compared with the preoperative values (95.6 ± 6.8%), while the other three groups showed no significant decrease. Due to their smaller physical characteristics, right liver donation was significantly more prevalent in the female groups than in the male groups (112/144, 77.8% vs 65/133, 48.9%; *p* < 0.001). The estimated liver resection rate correlated significantly with the splenic hypertrophy ratio (r = 0.528, *p* < 0.001) and the extent of the platelet count decrease (r = − 0.314, p < 0.001), but donor age and sex did not affect these parameters.

**Conclusion:**

Elderly female donors have a higher risk of postoperative skeletal muscle depletion. Additionally, female donors are more likely to donate a right liver graft, whose potential subclinical risks include postoperative splenic enlargement and a platelet count decrease.

## Background

Living-donor liver transplantation (LDLT) is an established treatment for end-stage liver disease or hepatocellular carcinoma with impaired liver function, especially in a country such as Japan where the shortage of deceased donor livers is a serious problem [1]. While LDLT initially began with paediatric liver transplant using the left lateral sector, the indications for LDLT have largely expanded to adult-to-adult liver transplantation using a left liver graft [2] or right liver graft, and the right liver is now the standard graft choice [3].

Donor safety is of the utmost importance in LDLT. For adult-to-adult liver transplantation, a right liver graft is preferred to a left liver graft due to the larger size of the right liver. Further, compared with the left liver, the anatomy of the hepatic hilum in the right liver is easier to divide and reconstruct [4]. It is well documented, however, that, compared with the left liver, procurement of the right liver results in a greater incidence of morbidity and mortality [5, 6], and is more invasive for the donor than the procurement of other graft types. In addition, donor surgeries are associated with serious ethical issues because of the inherent surgical stress and burden for the donors, both during and after donor surgery. Therefore, we [7] and other Japanese centres [8] believe that right liver procurement should not be the first choice as long as a sufficiently sized graft can be obtained with a left liver graft or right lateral sector graft. Few reports, however, provide the overall picture of the actual impact of graft procurement on a donor’s physical status.

Several recent reports have discussed postoperative depletion of skeletal muscle resulting from the surgical stress of LDLT donation [9] and other abdominopelvic surgery [10–12] . Others have reported an increase in the spleen volume and a decrease in the platelet count after live liver donation [13–16]. Therefore, we hypothesized that perioperative changes in such markers could be an indicator of surgical stress in liver donation, and investigated the actual impact of donor surgery with a focus on the postoperative skeletal muscle mass, spleen volume, and platelet count.

## Methods

### Study population

This retrospective study of prospectively collected data was conducted in accordance with the ethical guidelines for clinical studies at the Tokyo University Hospital. A total of 450 consecutive donor hepatectomies for LDLT were performed at the Department of Surgery, Artificial Organ and Transplantation Division, Tokyo University Hospital, from April 2001 through March 2017. All donors had undergone and passed our donor workup protocol, the details of which are described elsewhere [17], and were considered free from any comorbidities. Because the aim of this study was to evaluate the impact of donor surgery on the postoperative skeletal muscle mass, spleen volume, and platelet count, and it is well known that remnant liver regeneration and the recovery of laboratory data reaches a plateau within 3 months after surgery [18], donors who did not complete computed tomography (CT) imaging between postoperative months 2 and 4 were excluded from the study. Age of 45 years or older was defined as a clinically relevant cut-off value for elderly donors in accordance with an earlier study [19].

### Donor hepatectomy surgical technique

Our graft selection criteria and the technical details of our LDLT donor hepatectomies are described in previous reports [7, 17, 20, 21]. Basically, the graft should be over 40% of the standard liver volume [7, 17] of the recipient, and the left liver is the first choice when it satisfies the volume requirement. The subjects comprised 152 cases of right liver graft in which the main trunk of the middle hepatic vein (MHV) was preserved in the remnant donor left liver, named a right liver graft (RLG); 25 cases of extended right liver graft (ERLG), which includes the main trunk of the MHV on the graft side; 9 cases of left liver graft (LLG); 77 cases of left liver graft with the caudate lobe (LLG + CL; and 14 cases of right lateral sector graft (RLSG). In RLG procurement, venous tributaries of V5 and V8 in the graft were reconstructed as needed using cryopreserved venous allografts [20]. These graft variations were classified into two types: donors who underwent the RLG or ERLG procurement were categorized as the RL group (*n* = 177), and donors who underwent the LLG, LLG + CL, or RSLG procurement were categorized as the Non-RL group (*n* = 100).

The postoperative surgical complications were classified according to the Clavien-Dindo classification [22] and compared.

### Analysis of preoperative and postoperative skeletal muscle mass

**Fig. 1:**
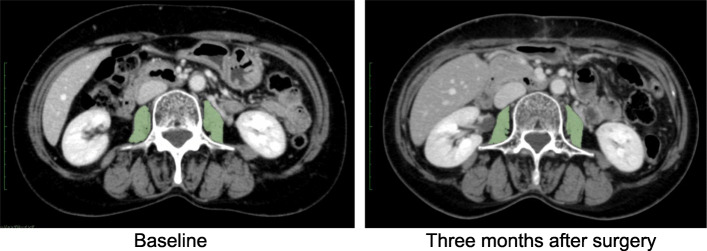
Representative case of postoperative muscular depletion. The green coloured area indicates the cross-sectional area of the psoas major muscle at the level of the third lumbar vertebrae

Skeletal muscle mass was measured on preoperative and postoperative CT images. We used the cross-sectional area of the psoas major muscle as an alternative to the skeletal muscle mass of the whole body in accordance with previous studies [9, 10, 23] The cross-sectional area of the bilateral psoas major muscles was named the total psoas muscle area (TPA, cm^2^). TPA was derived by measuring in a semi-automated manner the area included in a manual outline of the borders of the bilateral psoas major muscles with attenuation ranging from − 30 to 110 Hounsfield Units (HU) to exclude the vasculature and fatty infiltration at the level of the third lumbar (L3) vertebrae where both transverse processes are clearly visible. The TPA measurement was performed using image analysis software (OsiriX MD, Geneva, Switzerland). The TPA value was subsequently normalized to body size by dividing TPA by the square of body height (m^2^) [9, 10, 23], and the resulting value was defined as the total psoas index (TPI, cm^2^/m^2^). The rate of postoperative skeletal muscle depletion was calculated by dividing the preoperative TPI by the postoperative TPI. Representative preoperative and postoperative images are shown in Fig. 1.

### Volumetric analysis of the liver and spleen

Preoperative total liver volume (TLV) and spleen volume (SV_pre_) were measured from CT images obtained for the purpose of preoperative assessment using three-dimensional volume analysis software (SYNAPSE VINCENT, Fujifilm Inc., Tokyo, Japan). The ratio of TLV to SV_pre_ was calculated and defined as the liver/spleen ratio (LS ratio).

The extent of donor hepatectomy was calculated as follows. The actual weight of the graft liver was measured just after the perfusion of ice-cold lactate Ringer solution and subsequent University of Wisconsin cold storage solution in the back-table operation. It is next to impossible to measure actual graft volume, and therefore the actual graft weight was converted to the estimated graft volume (EGV) assuming that a graft weight of 1 g equals a volume of 1 ml. The estimated liver resection rate (ELRR) was calculated according to the following equation:

$$ \mathrm{ELRR}\left(\%\right)=\mathrm{EGV}\times 100/\mathrm{TLV} $$

Postoperative total spleen volume (SV_post_) was also measured as described above, and the splenic hypertrophy ratio (SHR) was calculated according to the following equation:

$$ \mathrm{SHR}\left(\%\right)={\mathrm{SV}}_{\mathrm{post}}\times 100/{\mathrm{SV}}_{\mathrm{pre}} $$

### Statistical analysis

Continuous valuables are expressed as means and standard deviations, and were compared using Student’s *t* test. Categorical variables were compared using the chi-square or Fisher’s exact test as appropriate. Logistic regression analysis was used for multivariate analysis to detect independent risk factors for a postoperative skeletal mass decrease, splenic hypertrophy, and platelet depletion. The cut-off values for these parameters were determined by selecting nice, rounded value closest to the quintile or quartile values respectively. Statistical significance was defined as *p* < 0.05. All statistical analyses were performed using SPSS statistical software (ver25.0 for Windows and Mac; Chicago, IL, USA).

## Results

### Study population

A total of 277 donors were included in this analysis. Of the 133 male donors and 144 female donors, 85 donors aged 45 or older were categorised as the elderly donor group, and the remaining 192 donors younger than 45 were categorised as the young donor group. Among the 277 donors, 177 were categorised as the RL group and the remaining 100 donors were categorised as the Non-RL group. The relationship of the donor to the recipient was as follows: son or daughter (*n* = 124), spouse (*n* = 72), sibling (*n* = 52), parent (*n* = 18), and other relation (*n* = 11).

### Perioperative factors of the donor population

**Fig. 2:**
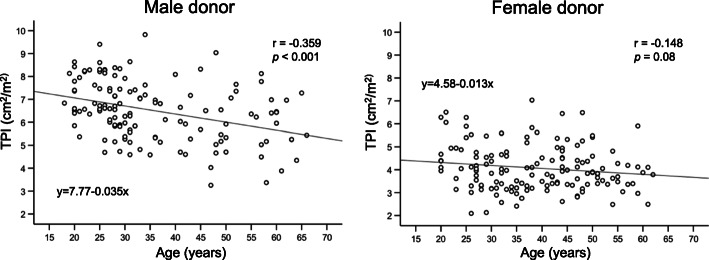
The sex-segregated correlation of donor age and the preoperative total psoas index. r: Pearson’s correlation coefficient; TPI: Total psoas index

Table 1 shows the body profiles, preoperative volumes of the liver and spleen, preoperative laboratory data, intraoperative variables, graft size, and postoperative variables of all donors and sex-segregated comparisons. Fundamental pre-existing sex differences in relation to physical size were detected. Body height, body weight, body mass index, body surface area, and all size-related parameters were significantly higher in men than in women. Preoperative TPA differed significantly between women and men, and this difference remained after adjusting for body size using TPI. TPI was significantly lower in women than in men (4.07 ± 0.99 cm^2^/m^2^ vs. 6.50 ± 1.29 cm^2^/m^2^). In the male donor group, donor age correlated significantly with TPI [Pearson’s correlation coefficient (r) = − 0.359, *p* < 0.001], but this correlation was not significant in women (r = − 0.148, *p* = 0.08) (Fig. 2). Both TLV and SV_pre_ were significantly smaller in women than in men, reflecting their physical characteristics. No significant difference in the LS ratio was detected between women and men.

**Table 1:** Donor profiles stratified by sex

	All donors *n* = 277	Women *n* = 144	Men *n* = 133	*p* value
**Recipient characteristics**				
Age (years)	37.5 ± 12.3	39.0 ± 11.2	36.0 ± 13.2	0.04
Height (cm)	165.1 ± 8.7	158.9 ± 5.4	171.7 ± 6.3	< 0.001
Weight (kg)	58.8 ± 10.4	52.3 ± 7.3	65 ± 88.5	< 0.001
BMI	21.5 ± 2.6	20.7 ± 2.6	22.3 ± 2.3	< 0.001
Body surface area (m^2^)	1.64 ± 0.17	1.52 ± 0.11	1.77 ± 0.12	< 0.001
Preoperative TPA (cm^2^)	14.55 ± 5.54	10.27 ± 2.46	19.2 ± 4.01	< 0.001
Preoperative TPI (cm^2^/m^2^)	5.24 ± 1.67	4.07 ± 0.99	6.50 ± 1.29	< 0.001
TLV (ml)	1206 ± 200	1014 ± 147	1224 ± 179	< 0.001
SV_pre_(ml)	136 ± 50	122 ± 43	150 ± 53	< 0.001
Preoperative LS ratio	10.0 ± 4.2	9.9 ± 3.7	10.1 ± 4.6	0.72
Preoperative T-Bil (mg/dl)	0.84 ± 0.14	0.79 ± 0.30	0.91 ± 0.39	0.01
Preoperative Alb (g/dl)	4.44 ± 0.32	4.39 ± 0.32	4.51 ± 0.30	0.001
Preoperative AST (IU/L)	18.0 ± 4.8	16.9 ± 4.1	19.2 ± 5.2	< 0.001
Preoperative ALT (IU/L)	18.1 ± 10.0	14.1 ± 6.2	22.4 ± 11.4	< 0.001
Preoperative ICG-R15min (%)	5.44 ± 1.98	5.09 ± 1.98	5.83 ± 1.91	0.02
Preoperative Cre (mg/dl)	0.69 ± 0.14	0.59 ± 0.08	0.82 ± 0.10	< 0.001
Preoperative Hgb (g/dl)	14.0 ± 1.5	13.0 ± 1.1	15.1 ± 1.0	< 0.001
Preoperative Plt (× 10^4^/μl)	25.2 ± 5.6	26.1 ± 5.9	24.3 ± 5.2	0.01
**Graft characteristics**				
Graft type RL / non-RL	177/100	112/32	65/68	< 0.001
Estimated liver resection rate (%)	45.9 ± 11.5	49.5 ± 10.2	41.9 ± 11.5	< 0.001
Actual graft weight (g)	540 ± 116	533 ± 100	549 ± 131	0.29
**Intra/postoperative data**				
Operative time (min)	464 ± 107	459 ± 133	469 ± 70	0.41
Estimated blood loss (g)	431 ± 252	384 ± 213	482 ± 281	0.001
EBL/kg (g/kg)	7.38 ± 4.23	7.42 ± 4.30	7.32 ± 4.16	0.83
Postoperative complication ≥ C-D 3b yes/no	6/269	2/142	4/129	0.43
Postoperative hospital stay (days)	14.2 ± 3.9	13.9 ± 3.9	14.5 ± 4.0	0.21

Data are presented mean ± standard deviations

*Abbreviations*: *Alb* albumin, *ALT* alanine aminotransferase, *AST* aspartate aminotransferase, *BMI* body mass index, *C-D* Clavien-Dindo, *Cre* creatinine, *EBL* estimated blood loss, *Hgb* haemoglobin, *ICG-R15* indocyanine green retention rate at 15 min, *LS ratio* liver/spleen ratio, *Plt* platelet count, *RL* right liver, *SV* splenic volume, *T-bil* total bilirubin, *TLV* total liver volume, *TPA* total psoas area, *TPI* total psoas index

The preoperative laboratory data were significantly different between women and men. Total bilirubin (T-bil), albumin (Alb), aspartate aminotransferase (AST), alanine aminotransferase (ALT), indocyanine green retention test at 15 min (ICG-R15), creatinine (Cre), and haemoglobin (Hgb) values were significantly higher in men than in women, while the platelet count was significantly higher in women than in men.

The ratio of right liver graft donation, i.e., the RL group, was 112/144 (77.8%) in women and 65/133 (48.9%) in men (*p* < 0.001). The ELRR was significantly higher in women than in men (49.5 ± 10.2% vs 41.9 ± 11.5%, p < 0.001), reflecting the graft type selection. The actual obtained graft weight, however, was similar between women and men (533 ± 100 g vs 549 ± 131 g, *p* = 0.29). There was no significant sex difference in the mean operation time or in the amount of blood loss (g/kg body weight).

With regard to donor surgical complications, no donor death or C-D classification grade greater 4a was observed. Six cases of C-D classification grade 3b were observed, all of which required re-laparotomy for postoperative major bile leakage. Rate of grade 3b complication and duration of postoperative hospital stay did not significantly differ between women and men.

Regarding factors associated with the prolonged hospital stay, major pre- and post-operative variables were analysed for the association, which revealed no significant risk factor except for the near significance of the elderly age (Table 2).

**Table 2:** Comparison of perioperative factors for prolonged postoperative hospital stay

	Prolonged postoperative hospital stay		
	≥ 20 days		
	Yes (n = 27)	No (*n* = 250)	*p* value
Age (years)	41.7 ± 14.6	37.1 ± 11.9	0.06
Age ≥ 45 / < 45 years	12 / 15	73 / 177	0.10
Sex Female / Male	15/ 12	118 / 132	0.41
Graft type RL / Non-RL	16 / 11	89 / 161	0.60
ELRR (%)	44.1 ± 11.8	46.0 ± 11.4	0.41
EBL per body weight > 10 ml/kg yes / no	3 / 24	58 / 192	0.15
Preoperative LS ratio < 7.0 yes / no	49 / 201	3 / 24	0.28
Preoperative Plt count < 20.0 × 10^4^/μl yes / no	2 / 25	42 / 208	0.27
Preoperative Alb level < 4.3 g/dl	10 / 17	65 / 185	0.22
Postoperative complication ≥ C-D 3b yes / no	1 / 26	5 / 245	0.56

*Abbreviations*: *Alb* albumin, *C-D* Clavien-Dindo, *EBL* estimated blood loss, *ELRR* estimated liver resection rate, *LS ratio* liver/spleen ratio, *Plt* platelet, *RL* right liver

### Postoperative skeletal muscle depletion

**Fig. 3:**
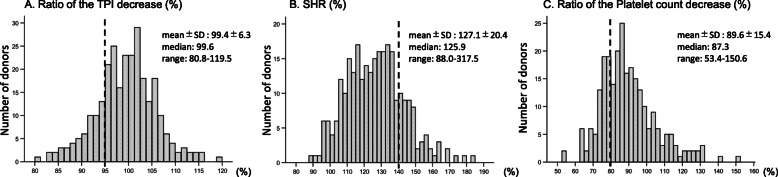
Distribution of the postoperative TPI decrease, SHR, and platelet count decrease. Dashed line indicates cut-off value. TPI: Total psoas index; SHR Splenic hypertrophy ratio

The distribution of the postoperative ratio of TPI to the preoperative value is shown in the histogram provided in Fig. 3a. The mean postoperative ratio of TPI for all donors was 99.3 ± 6.3%. The cut-off value for defining clinically relevant postoperative skeletal muscle depletion for the logistic regression model to detect risk factors was set as less than 95% of the preoperative TPI. The number of donors below the cut-off value was 59/277 (21.3%).

### Postoperative splenic hypertrophy

A histogram showing the distribution of the SHR of all donors is shown in Fig. 3b. We set the cut-off value for clinically relevant SHR as 140% for the logistic regression model, and 57/277 (20.5%) donors were beyond the cut-off value.

### Postoperative platelet count after liver donation

A histogram showing the distribution of the ratio of the postoperative platelet count to the preoperative count of all donors is presented in Fig. 3c. The cut-off value for a significant platelet count decrease was set as 80% for the logistic regression model. The ratio of donors whose values were below the cut-off value was 73/277 (26.4%).

### Factors associated with postoperative changes in the skeletal muscle mass, spleen volume, and platelet count

**Fig. 4:**
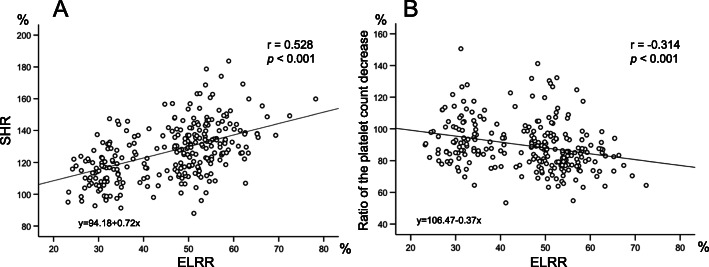
Correlation of the ELRR between the splenic hypertrophy ratio and postoperative platelet count decrease. r: Pearson’s correlation coefficient; ELRR: Estimated liver resection rate; SHR: Splenic hypertrophy ratio

Risk factors that influence the skeletal muscle depletion, splenic hypertrophy, and platelet count decrease were identified on the basis of univariate and multivariate analyses. Nine potential confounding factors were selected: elderly donor (≥ age 45), sex (female), graft type (RL group), high amount of estimated blood loss per body weight (≥10 ml/kg), long postoperative hospital stay (≥20 days), postoperative complication (presence of C-D classification ≥3b), preoperative low platelet count (< 20.0 × 10^4^/μl), preoperative low LS ratio, i.e., relatively large spleen to liver, (< 7.0), and preoperative low serum albumin (< 4.3 g/dl). Table 3 shows the results of the univariate and multivariate logistic regression analyses for a significant TPI decrease (< 95%), splenic hypertrophy (≥140%), and platelet depletion (< 80%). The analysis revealed that female and elderly age were both independent risk factors for postoperative skeletal muscle depletion (OR: 1.94, 95% C.I.:1.04–3.59 and OR: 2.30, 95% C.I. 1.27–4.24, respectively). The graft type, however, did not influence the skeletal muscle mass depletion. Univariate analysis revealed female donor and right liver graft procurement as significant factors for splenic hypertrophy, but multivariate analysis revealed that the actual independent risk factor for postoperative splenic hypertrophy was only a right liver graft (OR:7.30, 95% C.I. 2.76–19.34). Finally, with regard to platelet count decrease, right liver graft was an independent risk factor (OR: 3.24, 95% C.I. 1.69–6.21). The analysis revealed that a preoperatively low platelet count negatively affected the postoperative platelet count. After identifying the risk factors for postoperative skeletal muscle depletion, we compared donors who exhibited postoperative skeletal muscle depletion (< 95% of preoperative value) and those that did not (Table 4**)**. As shown in Table **4**, those with postoperative skeletal muscle depletion had a higher donor age and included a higher proportion of women than those who did not show postoperative muscle depletion. The proportion of donors with right liver procurement and the extent of the liver resection did not significantly differ between those with and without postoperative skeletal muscle depletion. The duration of the postoperative hospital stay was significantly longer in those with skeletal muscle depletion. Other factors, however, such as surgical complication greater than C-D classification 3b, splenic hypertrophy, and platelet count decrease, as well as postoperative haemoglobin level and serum albumin level had no significant effect on the postoperative skeletal muscle depletion. Consistent with previous reports, the ELRR significantly positively correlated with the SHR (r = 0.528, *p* < 0.001) and significantly negatively correlated with the ratio of the platelet count (r = − 0.314, *p* < 0.001) **(**Fig. 4**).**

**Table 3:** Clinical variables that influences total psoas index, splenic hypertrophy, and platelet count decrease

	Ratio of total psoas index decrease < 95%	Univariate analysis			Multivariate analysis			Splenic hypertrophy ratio ≥ 140%	Univariate analysis			Multivariate analysis			Ratio of platelet decrease < 80%	Univariate analysis			Multivariate analysis		
	*n* = 59	*p*	OR	95% C.I.	*p*	OR	95% C.I.	*n* = 57	*p*	OR	95% C.I.	*p*	OR	95% C.I.	*n* = 73	*p*	OR	95% C.I.	*p*	OR	95% C.I.
Sex																					
Female	39/144 (27.1%)	0.01	2.1	1.15–3.83	0.04	1.94	1.04–3.59	37/144 (25.7%)	0.03	1.96	1.07–3.58	0.42	1.3	0.69–2.47	36/144 (25.0%)	0.60	0.87	0.51–1.48			
Male	20/133 (15.0%)							20/133 (15.0%)							37/133 (27.8%)						
Age (years)																					
≥ 45	28/85 (32.9%)	0.02	2.55	1.41–4.62	0.01	2.3	1.27–4.24	18/85 (21.2%)	0.87	1.05	0.56–1.98				23/85 (31.5%)	0.86	1.05	0.59–1.88			
< 45	31/192 (16.1%)							39/192 (20.3%)							50/192 (26.0%)						
Graft type																					
RL	36/176 (20.3%)	0.60	0.86	0.47–1.55				52/177 (29.4%)	< 0.001	7.9	3.04–20.55	< 0.001	7.3	2.76–19.34	59/177 (33.3%)	< 0.001	3.07	1.61–5.86	< 0.001	3.24	1.69–6.21
Non-RL	23/100 (23.0%)							5/100 (5.0%)							14/100 (14.0%)						
Preoperative platelet count (×10^4^/μl)																					
< 20.0	10/44 (22.7%)	0.80	1.10	0.51–2.39				8/44 (18.2%)	0.67	0.83	0.36–1.91				5/44 (11.4%)	0.01	0.31	0.12–0.82	0.01	0.28	0.11–0.76
≥ 20.0	49/233 (21.0%)							49/233 (21.0%)							68/233 (29.2%)						
Preoperative LS ratio																					
< 7.0	8/52 (15.4%)	0.25	0.62	0.27–1.40				10/52 (19.2%)	0.79	0.9	0.42–1.93				15/52 (28.8%)	0.65	1.17	0.60–2.28			
≥ 7.0	51/225 (22.7%)							47/225 (20.9%)							58/225 (25.8%)						
Preoperative Alb (g/dl)																					
< 4.3	23/75 (30.7%)	0.02	2.04	1.11–3.75	0.18	1.54	0.81–2.91	15/75 (20.0%)	0.89	0.95	0.49–1.84				13/75 (17.3%)	0.04	0.5	0.25–0.97			
≥ 4.3	36/202 (17.8%)							42/202 (20.8%)							60/202 (29.7%)						
EBL per body weight (ml/kg)																					
≥ 10.0	12/61 (19.7%)	0.73	0.88	0.43–1.79				14/61 (24.6%)	0.60	1.2	0.61–2.38				16/61 (26.2%)	0.98	0.99	0.52–1.89			
< 10.0	47/216 (21.8%)							43/216 (19.9%)							57/216 (26.4%)						
Postoperative complication ≥ C-D 3b																					
yes	1/6 (16.7%)	1.00	0.73	0.08–6.41				3/6 (50.0%)	0.10	4.02	0.79–20.47				3/6 (50.0%)	0.19	2.87	0.57–15.56			
no	58/269 (21.2%)							54/271 (19.9%)							70/271 (26.8%)						
Postoperative hospital stay (days)																					
≥ 20	9/27 (33.3%)	0.11	2	0.85–4.72				5/27 (18.5%)	0.78	0.87	0.31–2.40				8/27 (29.6%)	0.65	1.2	0.50–2.87			
< 20	50/250 (20.0%)							52/250 (20.8%)							65/250 (26.0%)						

*Alb* albumin, *C-D* Clavien-Dindo, *C.I*. confidence interval, *EBL* estimated blood loss, *LS ratio* liver /spleen ratio, *OR* odds ratio, *RL* right liver

**Table 4:** Comparison of preoperative factors and postoperative outcomes between those with or without skeletal muscle depletion

	Postoperative skeletal muscle depletion		
	(TPI_post_ < 95% of TPI _pre_)		
	Yes (n = 59)	No (*n* = 218)	*p* value
Age (years)	40.8 ± 13.2	36.7 ± 11.9	0.02
Age ≥ 45 / < 45 years	28 / 31	57 / 161	0.002
Sex Female / Male	39 / 20	105 / 113	0.01
Graft type RL / Non-RL	36 / 23	141 / 77	0.60
ELRR (%)	44.3 ± 10.8	46.3 ± 11.6	0.25
EBL/kg (ml/kg)	7.60 ± 3.88	7.32 ± 4.32	0.65
Postoperative hospital stay (days)	15.2 ± 4.4	14.0 ± 3.8	0.03
Postoperative complication ≥ C-D 3b	1 / 58	5 / 213	1.00
Ratio of splenic hypertrophy (%)	124.5 ± 18.7	127.8 ± 20.9	0.28
Ratio of postoperative Hgb decrease (%)	94.8 ± 7.9	95.6 ± 8.4	0.54
Ratio of postoperative Plt decrease (%)	88.7 ± 13.6	89.9 ± 15.9	0.63
Ratio of postoperative Alb decrease (%)	95.1 ± 6.0	93.0 ± 6.6	0.74

*Abbreviations*: *Alb* albumin, *C-D* Clavien-Dindo, *EBL* estimated blood loss, *ELRR* estimated liver resection rate, *Hgb* haemoglobin, *Plt* platelet, *RL* right liver, *TPI* total psoas index

### Comparison of the extent of skeletal muscle depletion between sex and donor age

**Fig. 5:**
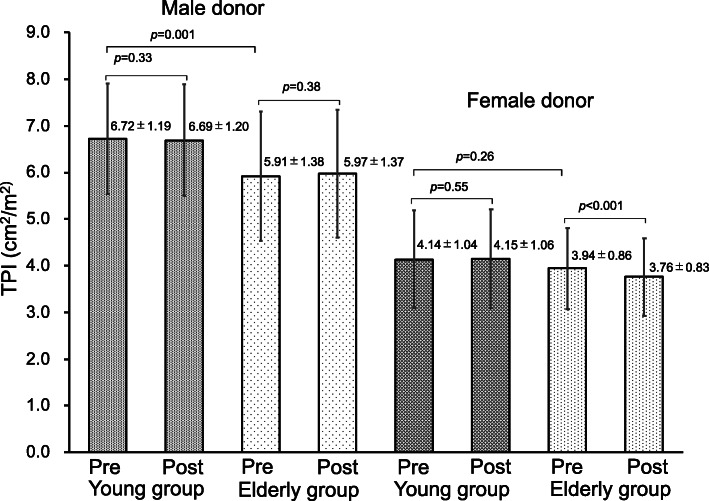
Change in the preoperative and postoperative TPI stratified by age group and sex. TPI: Total psoas index, Pre: preoperative, Post: postoperative

Preoperative and postoperative TPI were compared by stratifying all donors into four groups by sex and age (Fig. 5). Male donors showed a significant difference in the preoperative TPI between the elderly and young groups, but the postoperative skeletal muscle mass was almost unchanged in both groups. The preoperative and postoperative skeletal muscle mass also did not differ significantly in the young female group. Only the elderly female group showed a significant postoperative decrease in the TPI.

## Discussion

Ideally, there would be no aftereffects of liver procurement on the living donors because the LDLT donation is totally altruistic procedure, but more than a few physical and mental disadvantages of live liver donation in donors have been reported, both during and after surgery [5, 24, 25]. Unfortunately, there are also several reports of donor death [6, 26, 27]. Besides the operative morbidities and mortalities, transplant clinicians should be aware of even minimal aftereffects of hepatectomy on live donors even though it is subclinical, and live liver donor candidates should be informed of the details of such potential aftereffects. In this aspect, the present results showing the risk of skeletal muscular depletion among aged female donors and the splenic hypertrophy and accompanying platelet decrease after right liver donation will contribute to the future practice of LDLT.

The main aim of our study was to analyse the aftereffects of donor hepatectomies from the point of view of skeletal muscle depletion after donor hepatectomies and sex differences.

The preoperative TPI stratified by age was significantly lower in the male elderly group, but there was no significant difference in the preoperative TPI between young and elderly female groups. On the contrary, with regard to postoperative muscular depletion, only elderly female donors exhibited a significant decrease in skeletal muscle mass compared with the other three groups. Although we did not measure the testosterone or oestrogen levels in our preoperative assessment of the donor candidates, we speculate that the considerable postoperative skeletal muscle depletion observed in the elderly female donor group is due to oestrogen depletion. In general, oestrogen helps to maintain muscle mass in women and oestrogen levels decrease after menopause [28, 29]. In contrast, in men, testosterone is the main sex hormone that contributes to maintain muscle mass [30]. The definition of an elderly donor varies among previous reports, from 35 to 55 years of age [16, 19, 31]. In this study, we set the cut-off point at age 45. As a result, it is likely that mainly postmenopausal female donors would be assigned to the elderly group because the mean age of menopause is reported to be around 50 years of age [32], although we did not collect information about menstrual status in the present cohort.

To the best of our knowledge, this is the first study to analyse the effect of major hepatectomy focusing on skeletal muscle mass and discussing the age and sex differences among LDLT donors. Live donors for organ transplantation are fundamentally healthy because they must pass strict preoperative medical examinations. Our hospital strictly excluded any donor candidate who had history of comorbidities requiring medication or history of malignant diseases [17], therefore, changes in the skeletal muscle mass before and after donor surgery are purely attributable to surgical stress including the effect of hospitalization itself which is associated with reduced caloric intake, lack of exercise, or prolonged bed rest and can lead to decrease in muscle mass [12]. Pre-existing skeletal muscle depletion before surgery, termed sarcopenia, is now widely considered a postoperative risk factor in various surgical procedures. Several reports indicate that a preoperative status of sarcopenia negatively affects the postoperative prognosis of LDLT recipients [33, 34] as well as those undergoing other gastrointestinal or hepato-biliary-pancreatic surgeries [12, 23, 35] In recent years, there are also several studies that have compared changes in the preoperative and postoperative states of the skeletal muscle. Miyake et al. reported that, in addition to preoperative sarcopenia status, skeletal muscle loss greater than 10% after radical cystectomy for urothelial carcinoma of the bladder is associated with shorter overall survival [10]. Otuji et al. reported that postoperative skeletal muscle loss 7 days after major hepatectomy with extrahepatic bile duct resection is associated with postoperative morbidity and mortality [11]. Postoperative accelerated muscle loss after pancreatic resection negatively impacted survival in pancreatic cancer was also reported [12]. The fundamental difference between our report and these studies is that the subjects in the other studies had malignant disease, while our study focused on definitely healthy people, and therefore, our study eliminated the effect of cancer-induced cachexia [36]. In addition, essence of the donor hepatectomy is summarized to simple resection of the liver and it requires no gastrointestinal reconstruction, i.e. alteration of gastrointestinal structures, due to resection of the primary lesion of the malignancies, or postoperative adjuvant chemotherapy [12], that would affect the postoperative nutritional status.

The number of studies that focusing on muscle wasting after LDLT donation is very limited. Matsumoto reported that a bilateral subcostal incision with midline extension (Mercedes incision) for LDLT donation results in significantly greater atrophy of the rectus abdominis muscle compared with a simple midline incision [37]. They concluded that the muscular atrophy following the Mercedes incision is a result of cutting the dominant nerve of the rectus abdominis muscle. In contrast, to eliminate the direct effect of laparotomy, we measured the psoas major muscle because it locates on the dorsal part of the body and was isolated from the surgical site. Furthermore, using the psoas major muscle to evaluate the amount of skeletal muscle mass is an established method presented by several preceding studies [9, 33]. Only one preceding study that addressed the postoperative change of the psoas major muscle of the LDLT donor was reported [9] . This report showed that psoas muscle significantly decreased in both men and women during the first 1 week after surgery and then gradually and fully recovered to preoperative levels 3 months after donor surgery. This result complemented our study in terms of relatively acute postoperative period, such as first 1 week, because we did not investigate routinely postoperative CT in this acute period unless postoperative complication was suspected. In other words, according to this preceding report, the term 3 months is enough to complete almost recovery of the skeletal muscle wasting. And the detectable difference of skeletal muscle depletion 3 months after surgery would reflect the difference of resilience of each individual to donor surgery.

Secondary, our findings revealed that female donor provide right liver graft more often than male donor. Donation of the right liver is associated with a significant postoperative increase in the spleen volume and decrease in the platelet count. These phenomena are also described in several previous reports [13–16] . Here we confirmed in a relatively larger cohort that the degree of the postoperative splenic hypertrophy and thrombocytopenia correlated with the ELRR. The preceding study concluded that the extent of liver resection rate for donor surgery did not correlate with skeletal muscle depletion both 1 week and 3 months after donor surgery [9], although that study limited the subject to the donor who underwent right liver procurement only, therefore, the range of the liver resection rate is narrower than our study. The right liver procurement, which is essentially larger graft than left liver or right lateral sector graft procurement, was not risk factor for postoperative skeletal muscle depletion 3 months after surgery in our study strengthen the preceding result. Therefore, amount of the remnant liver, in the acceptable range to perform donor surgery safely, i.e. keeping the remnant liver larger than 30% of donor’s preoperative TLV, plays only a minor role to determine the degree of postsurgical skeletal muscle depletion.

Literatures regarding the impact the skeletal muscle depletion after surgery have been scarce. One study reported that the postoperative skeletal muscle depletion is associated with swallowing function after cardiovascular surgery [38]. The subjects of this study was much older patients of cardiovascular diseases who needed aggressive rehabilitation after operation, which makes it difficult to associate with the present study. Moreover, the minimal skeletal depletion among healthy donors is not likely to affect the postoperative conditions of donors. However, present results revealed that elderly female was at a higher risk of postoperative skeletal muscle depletion after operation even among healthy living donor, which might warrant the future studies investigating the possible sequelae of the skeletal muscle depletion after the operation among other populations.

From a clinical standpoint, the level of the change in the skeletal muscle mass 3 months after donor hepatectomy was relatively slight and subclinical and that is also compatible with the proceeding study [9]. And no obvious adverse effects related to skeletal muscle depletion were detected in our population; therefore, LDLT per se is feasible as ever, regardless of age or sex. Our findings, however, provide evidence of a certain amount of surgical stress on the donor and the responsiveness of each donor to these stresses differ from their age and sex. Transplant clinicians should pay careful attention to the personal characteristics of each individual and inform donor candidates of the possibility of future muscular weakness, especially in elderly females. In addition, we should be aware that right liver procurement induces subclinical portal hypertension in the donor, as demonstrated by the splenic hypertrophy and platelet decrease.

Our study has several limitations. First, this was a retrospective study in a single institution, and we excluded a certain number of donors due to the absence of postoperative CT images within the appropriate period. Second, the relation between skeletal muscle depletion and postoperative symptoms on the donor’s quality of life was not assessed and remains unclear. Finally, the observation period of this study was relatively short and aftereffects of hepatectomy on skeletal muscle mass over a longer period of time, i.e. 6 months, 1 year, or longer were not investigated. The possibility of further muscle depletion or the recovery of skeletal muscle mass requires further evaluation with a longer observation time.

## Conclusions

In conclusion, transplant surgeons should pay attention to the possibility of skeletal muscular depletion, even at subclinical levels, in aged female donors. In addition, female donors provide a right liver graft significantly more often than male donors because of their relatively smaller physical characteristics, which carries subclinical risks of postoperative splenic hypertrophy and platelet decrease. We hope that these results will facilitate donor selection and be useful for counselling future donor candidates.

## Data Availability

The datasets used and analysed during this study are available from the corresponding author upon reasonable request.

## Abbreviations

Alb: Serum albumin level
ALT: Alanine aminotransferase
AST: Aspartate aminotransferase
BMI: Body mass index
CT: Computed tomography
C-D: Clavien-Dindo
Cre: Creatinine
ELRR: Estimated liver resection rate
ICG-R15: Indocyanine green retention rate at 15 min
LDLT: Living donor liver transplantation
LS ratio: Liver/spleen ratio
Plt: Platelet count
SHR: Splenic hypertrophy ratio
SV: Spleen volume
T-bil: Total bilirubin
TLV: Total liver volume
TPA: Total psoas area
TPI: Total psoas index
